# Increasing sexually transmitted infection rates in young men having sex with men in the Netherlands, 2006–2012

**DOI:** 10.1186/1742-7622-11-12

**Published:** 2014-08-28

**Authors:** Femke DH Koedijk, Birgit HB van Benthem, Eliane MDC Vrolings, Wim Zuilhof, Marianne AB van der Sande

**Affiliations:** 1 Centre for Infectious Disease Control, RIVM National Institute of Public Health and the Environment, P.O. Box 1, 3720 BA Bilthoven, Utrecht, The Netherlands; 2 Municipal Health Service Twente, Enschede, The Netherlands; 3 STI AIDS Netherlands, Amsterdam, The Netherlands; 4 Julius Center for Health Sciences and Primary Care, University Medical Centre Utrecht, Utrecht, The Netherlands

**Keywords:** Commercial sex, Adolescents, Gay men, Epidemiology, Surveillance, Ethnicity

## Abstract

**Background:**

Men having sex with men (MSM) remain the largest high-risk group involved in on-going transmission of sexually transmitted infections (STI), including HIV, in the Netherlands. As risk behaviour may change with age, it is important to explore potential heterogeneity in risks by age. To improve our understanding of this epidemic, we analysed the prevalence of and risk factors for selected STI in MSM attending STI clinics in the Netherlands by age group.

**Methods:**

Analysis of data from the national STI surveillance system for the period 2006–2012. Selected STI were chlamydia, gonorrhoea, infectious syphilis and/or a new HIV infection. Logistic regression was used to identify factors associated with these selected STI and with overall STI positivity. Analyses were done separately for MSM aged younger than 25 years and MSM aged 25 years and older.

**Results:**

In young MSM a significant increase in positivity rate was seen over time (p < 0.01), mainly driven by increasing gonorrhoea diagnoses, while in MSM aged 25 and older a significant decrease was observed (p < 0.01). In multivariate analyses for young MSM, those who were involved in commercial sex were at higher risk (OR: 1.5, 95% CI: 1.2-1.9). For MSM aged 25 years and older this was not the case. Having a previous negative HIV test was protective among older MSM compared to those not tested for HIV before (OR: 0.8, 95% CI: 0.8-0.8), but not among younger MSM.

**Conclusions:**

MSM visiting STI clinics remain a high-risk group for STI infections and transmission, but are not a homogenous group. While in MSM aged older than 25 years, STI positivity rate is decreasing, positivity rate in young MSM increased over time. Therefore specific attention needs to be paid towards targeted counselling and reaching particular MSM sub-groups, taken into account different behavioural profiles.

## Background

Half of all new sexually transmitted infections (STI) occurs among young people aged 15–24 years [1,2]. High-risk groups drive STI transmission, with men having sex with men (MSM) being the largest single high-risk group in many industrialised countries. MSM are estimated to represent 5-9% of the male population in the Netherlands [3], and are responsible for 40% of consultations in men at Dutch STI clinics [1].

Young MSM are of special public health importance for STI and Human Immunodeficiency Virus (HIV) prevention worldwide. Young MSM often report participation in high-risk sexual behaviour and considerable numbers of HIV infections have been observed in several studies [4-6]. A recent European study showed that in the last decade, the largest increase of new HIV infections has been seen among young MSM [7]. Young MSM are at significantly greater risk of HIV seroconversion than older MSM [8]. Risk behaviour may have increased due to treatment optimism [9,10], which can partly offset the gains made with safe sex campaigns. A recent Dutch study [11] showed that young MSM visiting STI clinics were at higher risk for both a single STI infection and STI co-infections than older MSM. However, that study did not go into detail about specific risk factors for young and older MSM. Here we analyse trends in different STI and HIV prevalence among MSM visiting STI clinics of two age-groups (MSM aged under 25 years and MSM aged 25 years and older), focusing on underlying risk factors and risk behaviour. The results can be used to guide targeted control strategies.

## Methods

Since 2006, 26 dedicated STI clinics, mostly within the municipal public health services and some of them with additional outside test locations, provide anonymous low threshold STI/HIV testing and care, which is free of charge and targeted at high-risk groups [1]. This system of test and treat with follow up care was set up in addition to the regular national health services, to reach people who might otherwise not seek STI care timely. Persons matching one of the following criteria are considered to be at high-risk of STI acquisition: (1) reporting STI-related symptoms, (2) notified or referred for STI testing, (3) aged below 25 years, (4) men having sex with men (MSM), (5) involved in commercial sex, (6) originating from an HIV/STI endemic area, (7) reporting three or more sexual partners in the previous six months or (8) reporting a partner from one of these risk groups. All consultations and corresponding diagnoses are reported to the Centre for Infectious Disease Control (RIVM) for surveillance purposes, facilitated by a web based application (SOAP). The unit of analysis is ‘new STI consultation’, which is defined as a consultation for new symptoms or one resulting from routine STI screening of asymptomatic cases, both involving laboratory testing and medical examination. At each consultation, information on demographics (gender, year of birth, ethnicity), behaviour (STI history, commercial sex work (CSW)), diagnostics and clinical outcome is recorded by the clinician. All visitors were asked whether they had sex with men, women or both in the past six months. In this study, an MSM was defined as a men who reported to have had sex with men (and women) in the past six months. All clients are tested for chlamydia, gonorrhoea and syphilis and since 2010 an opt-out policy for HIV testing has been implemented. Other STI are tested on indication. Microbiological diagnostics are carried out locally at laboratories related to the STI clinics in accordance with standard procedures established in an STI screening protocol [12], including quality control measures.

### Statistical analyses

For each STI (chlamydia, gonorrhoea, syphilis and HIV) the positivity rate was calculated by dividing the number of infections by the number of tests performed for that specific STI. Overall STI positivity rate was calculated by dividing the number of consultations in which at least one STI was found, by the total number of consultations. The Chi-square test was used for testing differences in proportions and trends in STI prevalence were determined using the Chi-square test for trend. A p-value <0.05 was considered to be significant. Univariate regression analyses were performed to identify factors associated with an infection. Factors associated with an STI with a p-value <0.20 were further analysed by backward multivariate logistic regression. In the multivariable model, variables with p < 0.01 were considered statistically significant. Analyses were performed per STI and also for the overall risk on an STI. Analyses were split up by age group: younger than 25 years and 25 years or older. Known HIV positive MSM were excluded from the analyses, since they are known to have a very different risk profile. Analyses were carried out using the SAS software version 9.2.

## Results

### Characteristics

**Figure 1:**
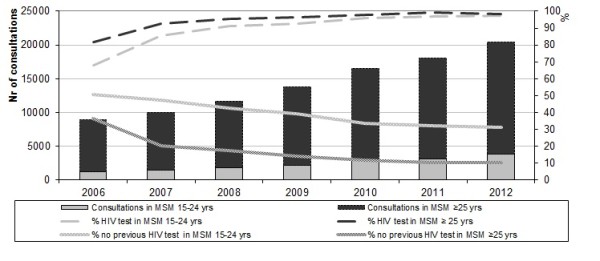
Trends in STI consultations and HIV test behaviour for MSM visiting STI clinics, by age group, the Netherlands, 2006–2012.

Between 2006 and 2012, 99,105 new STI consultations in MSM were registered in the national database at the RIVM. Of these, 17% (n = 16,603) were in MSM younger than 25 years of age (Table 1) and 83% was 25 years and older. The number of consultations in MSM visiting an STI clinic increased steadily since 2006 (Figure 1). The number of consultations in MSM aged under 25 increased with 218% from 1,191 in 2006 to 3,791 in 2012; this increase was 114% in those aged 25 and older; from 7,761 in 2006 to 16,633 in 2012.

**Table 1:** Socio-demographic and behavioural characteristics and STI (chlamydia, gonorrhoea, syphilis or HIV) positivity rate by age group in consultations in MSM visiting STI clinics, the Netherlands, 2006-2012

**Characteristics**	**15-24 yrs (n = 16,603; STI positivity =18.6%)**		**≥ 25 yrs (n = 82,502; STI positivity =17.7%)**	
	**N (%)**	**STI positivity (%)**	**N (%)**	**STI positivity (%)**
**Age, years**				
15-19	3023 (18.2)	16.4		
20-24	13580 (81.8)	19.1		
25-34			28550 (34.6)	19.5
35-44			26932 (32.6)	18.3
45-54			17871 (21.7)	16.5
≥55			9149 (11.1)	12.9
**Sexual behaviour**				
Sex with both men and women	2700 (16.3)	16.4	16391 (19.9)	12.9
Sex with men only	13903 (83.7)	19.0	66111 (80.1)	18.9
**Ethnicity**				
Dutch	12657 (76.2)	17.0	64657 (78.4)	17.0
Surinam/Antilles	854 (5.1)	28.3	2351 (2.9)	23.5
Turkey/Morocco	373 (2.3)	19.6	1291 (1.6)	20.4
Eastern Europe	486 (2.9)	27.6	1425 (1.7)	21.7
Sub-Sahara Africa	167 (1.0)	23.4	449 (0.5)	24.3
Latin America	359 (2.2)	29.8	1871 (2.3)	22.0
Asia	551 (3.3)	18.5	2633 (3.2)	22.1
Other	1156 (7.0)	20.9	7825 (9.5)	18.2
**Previous HIV test**				
No	6123 (36.9)	16.5	12785 (15.5)	18.8
Yes	10043 (60.5)	20.0	67622 (82.0)	17.6
Unknown	437 (2.6)	13.7	2095 (2.5)	16.2
**Previous STI**				
No	12618 (76.0)	17.4	60215 (73.0)	16.1
Yes	1915 (11.5)	28.8	12150 (14.7)	24.9
Unknown	2070 (12.5)	16.2	10137 (12.3)	18.6
**CSW**				
No	15822 (95.3)	18.3	80153 (97.2)	17.8
Yes, in past 6 months	592 (3.6)	29.7	1386 (1.7)	16.9
Unknown	189 (1.1)	7.4	962 (1.2)	10.3
**Client of CSW**				
No	16281 (98.1)	18.7	79640 (96.5)	18.0
Yes, in past 6 months	149 (0.9)	18.8	1988 (2.4)	9.5
Unknown	173 (1.0)	8.7	874 (1.1)	12.0
**Nr of partners in < 6 months**				
0-1	2884 (17.4)	13.6	8342 (10.1)	12.9
2-10	9581 (57.7)	18.9	40959 (49.7)	17.1
>10	992 (6.0)	31.1	10751 (13.0)	20.4
Unknown	3146 (18.9)	19.0	22450 (27.2)	19.4
**STI symptoms**				
No	11134 (67.1)	14.9	50728 (61.5)	13.6
Yes	3076 (18.5)	34.0	17607 (21.3)	28.7
Unknown	2393 (14.4)	16.1	14167 (17.2)	19.0
**Notified**				
No	12239 (73.7)	16.2	58147 (70.5)	15.5
Yes	1992 (12.0)	36.0	9764 (11.8)	29.0
Unknown	2372 (14.3)	16.3	14591 (17.7)	19.4
**STI positivity**				
Chlamydia	1627 (9.9)	-	7292 (8.9)	-
Gonorrhoea	1465 (9.0)	-	6078 (7.5)	-
Infectious syphilis	272 (1.7)	-	1867 (2.3)	-
HIV	246 (1.5)	-	1778 (2.4)	-

As shown in Table 1, MSM visiting STI clinics aged younger than 25 years reported more often having sex exclusively with other men, were less often of native Dutch, reported less often to be tested for HIV before, reported less often to have had a previous STI, reported more often to be involved in commercial sex work, were less often client of sex workers, reported less often to have had 10 or more sexual partners in the previous six months and reported less often STI symptoms than MSM aged 25 years or older (all p < 0.01). In both age groups, 12% reported to be notified for an STI.

### STI positivity rates

The overall STI positivity rate was significantly higher in young MSM (15–24 years), compared to MSM aged 25 years or older; 18.6% versus 17.7%; (p < 0.01). In both age groups, men having sex with men exclusively had a significantly higher STI positivity rate than men having sex with both men and women (p < 0.01). In young MSM, positivity rate was highest in men originating from Latin America (30%), in MSM aged 25 or older it was highest among MSM from Surinam and the Dutch Antilles and Sub-Sahara Africa (both 24%). In both age groups, STI positivity rate was highest in those who reported a previous STI in the past two years, multiple sex partners, STI symptoms and to be notified by a partner for an STI. Positivity rate in MSM under 25 years of age who were involved in CSW was 30%, which was significantly higher than in MSM of 25 years and older involved in CSW (17%; p < 0.01). Positivity rates for chlamydia and gonorrhoea were significantly higher in MSM younger than 25 years compared MSM aged 25 years and older, while syphilis and HIV positivity rates were higher in MSM aged 25 years and older (all p < 0.01).

### Time trends in positivity rates by age group

**Figure 2:**
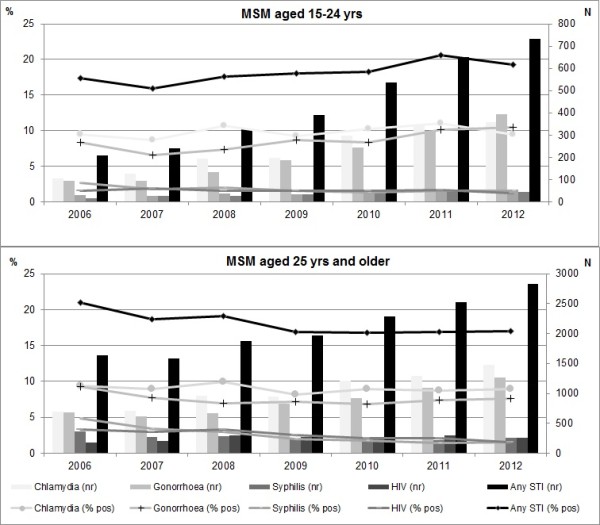
Trends in STI positivity rate (left axis) and number of STI (right axis) for different STI for MSM visiting STI clinics, by age group, the Netherlands, 2006–2012.

As shown in Figure 2, an increasing trend in STI positivity over time was observed in MSM younger than 25 years; from 17% and 16% in 2006 and 2007 to 21% in 2011 and 19% in 2012 (p < 0.01). In MSM aged 25 years and older a significant decrease was seen: from 22% in 2006 to 17% in 2009 and subsequent years (p < 0.01). The increasing positivity rate in young MSM was mainly caused by increasing positivity rates for gonorrhoea, which increased significantly from 8.4% in 2006 to 10.5% in 2012 (p < 0.01). In MSM aged 25 years and older, a significant decreasing trend in positivity rate was found for all STI except for chlamydia, which remained stable.

### Logistic regression analyses for young MSM

Table 2 presents the results for multivariable logistic regression analyses for the characteristics associated with having at least one STI among MSM younger than 25 years. An additional file shows a table with the univariate analyses for any STI [see Additional file 1].

**Table 2:** Multivariate regression analyses for any STI and per STI in consultations in young MSM (<25 years) visiting STI clinics, the Netherlands, 2006-2012

	**Any STI OR (95% CI)**	**Chlamydia OR (95% CI)**	**Gonorrhoea OR (95% CI)**	**Infectious syphilis OR (95% CI)**	**HIV OR (95% CI)**
**Year of consult**	NS	NT	**1.1 (1.0-1.1)**	NS	NT
**Age, years**					
15-19	NS	NT	NS	NS	1.0
20-24					**1.8 (1.1-2.7)**
**Sexual behaviour**					
Sex with both men and women	1.0	NT	1.0	NS	NS
Sex with men only	**1.3 (1.2-1.5)**		**1.6 (1.4-1.9)**		
**Ethnicity**					
Dutch	1.0	1.0	1.0	1.0	1.0
Surinam/Antilles	**1.9 (1.6-2.2)**	**1.5 (1.2-1.9)**	**1.6 (1.3-2.0)**	**2.5 (1.6-3.7)**	**2.8 (1.8-4.3)**
Turkey/Morocco	1.2 (0.9-1.6)	0.9 (0.6-1.3)	1.2 (0.8-1.7)	1.3 (0.6-2.9)	1.9 (0.9-4.0)
Eastern Europe	**1.5 (1.2-1.9)**	1.2 (0.9-1.7)	1.0 (0.8-1.5)	**2.7 (1.7-4.5)**	**3.1 (1.9-5.1)**
SS Africa	**1.5 (1.1-2.2)**	1.3 (0.8-2.1)	1.3 (0.8-2.2)	2.0 (0.7-5.4)	**5.1 (2.5-10.4)**
Latin America	**1.9 (1.5-2.4)**	1.3 (0.9-1.8)	**1.8 (1.3-2.4)**	**3.1 (1.8-5.4)**	**4.5 (2.7-7.5)**
Asia	1.1 (0.9-1.3)	0.9 (0.7-1.2)	1.0 (0.8-1.5)	1.5 (0.8-2.9)	0.9 (0.4-2.1)
Other	**1.3 (1.1-1.5)**	0.9 (0.8-1.2)	**1.2 (1.0-1.5)**	1.5 (1.0-2.4)	**2.1 (1.4-3.3)**
**Previous HIV test**					
No	NS	NS	NS	1.0	1.0
Yes, negative				1.0 (0.8-1.3)	0.8 (0.6-1.0)
Unknown				**2.8 (1.5-5.4)**	**2.6 (1.4-2.8)**
**Previous STI**					
No	1.0	1.0	1.0	1.0	1.0
Yes	**1.8 (1.6-2.0)**	**1.5 (1.3-1.7)**	**1.8 (1.5-2.1)**	**1.9 (1.4-2.6)**	**2.0 (1.4-2.8)**
Unknown	1.0 (0.9-1.2)	1.0 (0.9-1.3)	0.9 (0.7-1.1)	0.5 (0.3-0.8)	0.8 (0.5-1.3)
**CSW**					
No	1.0	1.0	1.0	1.0	NS
Yes, in <6 months	**1.5 (1.2-1.9)**	**1.4 (1.1-1.8)**	**1.5 (1.1-2.0)**	**2.7 (1.8-4.2)**	
Unknown	**0.4 (0.2-0.7)**	**0.5 (0.2-0.9)**	0.6 (0.3-1.2)	-	
**Client of CSW**					
No	NS	NS	NT	NT	NT
Yes, in <6 months					
Unknown					
**No of partners in < 6 months**					
0-1	1.0	1.0	1.0	NS	1.0
2-10	**1.4 (1.3-1.6)**	**1.3 (1.1-1.5)**	**1.6 (1.3-1.9)**		1.1 (0.7-1.7)
>10	**2.3 (1.9-2.8)**	**2.0 (1.5-2.5)**	**2.5 (2.0-3.2)**		**3.7 (2.3-6.0)**
Unknown	**1.4 (1.2-1.7)**	1.2 (1.0-1.5)	**1.9 (1.5-2.3)**		1.2 (0.7-2.0)
**Notified**					
No	1.0	1.0	1.0	1.0	1.0
Yes	**2.9 (2.6-3.2)**	**2.4 (2.1-2.7)**	**2.9 (2.5-3.3)**	**2.3 (1.7-3.1)**	**1.9 (1.4-2.6)**
Unknown	1.1 (0.9-1.3)	1.1 (0.9-1.3)	**1.3 (1.0-1.6)**	**2.1 (1.5-3.2)**	1.3 (0.8-2.1)

NS: Not significant. NT: Not tested in multivariate model (p >0.20 in univariate model).

In bold: significant (p < 0.01); due to rounding into 1 decimal 1.0 is not always significant.

Young MSM who reported a previous STI or being notified for an STI were at higher risk on having a new STI compared to those who did not report this. Ethnicity was also associated with the risk of testing positive for STIs, but this differed per STI: for chlamydia only those from Suriname or the Dutch Antilles were at significant higher risk compared to young native Dutch MSM (OR 1.5; 95% CI: 1.2-1.9), while for HIV almost all non-Dutch young MSM were at higher risk. MSM younger than 25 years from Latin America were at significant higher risk for gonorrhoea (OR 1.8), syphilis (OR 3.1) and HIV (OR 4.5).

MSM younger than 25 years who reported to be involved in commercial sex work (CSW) were at significantly higher risk for chlamydia (OR 1.4), gonorrhoea (OR 1.5) and syphilis (OR 2.7) compared to those not involved in CSW. Furthermore, the number of sex partners was also significantly associated with increased risk for all STI except for syphilis.

### Logistic regression analyses for older MSM

Table 3 is showing the results of the multivariate analyses for any STI and for all STI separately. In MSM aged 25 and older, having sex with men exclusively (overall OR 1.5), a previous STI (OR 1.7), and being notified for an STI (OR 2.2) were independent risk factors associated with all STI. An additional file shows a table with the univariate analyses for any STI [see Additional file 1]. Those MSM who had been tested negative for HIV at a previous visit to the STI clinic were at significantly lower risk than those not tested for HIV previously (OR 0.8). Similar with MSM younger than 25 years, older MSM clinic visitors from Suriname or the Dutch Antilles were at higher risk for all STI compared to Dutch MSM (overall OR 1.5). All non-Dutch MSM visitors, except those from Turkey or Morocco, were at higher risk for testing HIV positive compared to Dutch MSM, with those from Sub Saharan Africa being at highest risk (OR 3.8). For chlamydia and gonorrhoea, risk on positivity decreased significantly with age, while risk on syphilis increased with age. As in young MSM, reporting an increasing number of sexual partners was significantly associated with risk on testing positive for all STI, except for syphilis.

**Table 3:** Multivariate regression analyses for any STI and per STI in MSM aged 25 or older visiting STI clinics, the Netherlands, 2006-2012

	**Any STI OR (95% CI)**	**Chlamydia OR (95% CI)**	**Gonorrhoea OR (95% CI)**	**Infectious syphilis OR (95% CI)**	**HIV OR (95% CI)**
**Year of consult**	**0.97 (0.95-0.98**	NS	NS	**0.8 (0.8-0.9)**	**0.8 (0.8-0.9)**
**Age, years**					
25-34	1.0	1.0	1.0	1.0	NS
35-44	**0.9 (0.9-1.0)**	**0.9 (0.9-1.0)**	**0.9 (0.8-0.9)**	**1.3 (1.2-1.4)**	
45-54	**0.9 (0.8-0.9)**	**0.9 (0.8-0.9)**	**0.7 (0.6-0.7)**	**1.6 (1.4-1.8)**	
>54	**0.7 (0.6-0.7)**	**0.8 (0.7-0.8)**	**0.5 (0.4-0.5)**	**1.5 (1.2-1.7)**	
**Sexual behaviour**					
Sex with both men and women	1.0	1.0	1.0	1.0	1.0
Sex with men only	**1.5 (1.4-1.6)**	**1.3 (1.2-1.4)**	**1.7 (1.5-1.8)**	**1.5 (1.3-1.7)**	**2.0 (1.7-2.4)**
**Ethnicity**					
Dutch	1.0	1.0	1.0	1.0	1.0
Surinam/Antilles	**1.5 (1.3-1.6)**	**1.2 (1.1-1.4)**	**1.3 (1.1-1.5)**	**2.0 (1.6-2.5)**	**2.3 (1.9-2.9)**
Turkey/Morocco	**1.2 (1.1-1.4)**	**1.2 (1.0-1.5)**	**1.3 (1.1-1.5)**	1.1 (0.7-1.7)	1.4 (1.0-2.0)
Eastern Europe	**1.3 (1.1-1.4)**	1.1 (0.9-1.3)	1.2 (1.0-1.4)	1.0 (0.7-1.6)	**2.2 (1.7-2.9)**
SS Africa	**1.6 (1.3-2.0)**	**1.4 (1.0-1.9)**	**1.4 (1.0-1.9)**	1.0 (0.5-1.9)	**3.8 (2.5- 5.7)**
Latin America	**1.3 (1.1-1.4)**	1.0 (0.9-1.2)	1.1 (1.0-1.3)	**1.7 (1.3-2.2)**	**2.7 (2.1-3.4)**
Asia	**1.3 (1.2-1.4)**	1.3 (1.1-1.4)	**1.2 (1.0-1.4)**	1.3 (1.0-1.7)	**1.5 (1.2-1.9)**
Other	1.0 (1.0-1.1)	0.9 (0.8-1.0)	1.0 (1.0-1.1)	1.0 (0.9-1.2)	**1.4 (1.2-1.6)**
**Previous HIV test**					
No	1.0	1.0	1.0	1.0	1.0
Yes, negative	**0.8 (0.8-0.8)**	**0.8 (0.7-0.9)**	**0.9 (0.8-1.0)**	**0.7 (0.6-0.7)**	**0.6 (0.5-0.7)**
Unknown	**0.8 (0.7-0.9)**	**0.8 (0.6-0.9)**	0.8 (0.6-0.9)	1.0 (0.8-1.4)	1.2 (0.9-1.5)
**Previous STI**					
No	1.0	1.0	1.0	1.0	1.0
Yes	**1.7 (1.6-1.7)**	**1.5 (1.4-1.6)**	**1.6 (1.5-1.8)**	**1.5 (1.3-1.7)**	**2.0 (1.4-2.8)**
Unknown	1.0 (0.9-1.1)	1.0 (0.9-1.1)	**1.1 (1.0-1.3)**	**0.8 (0.7-1.0)**	0.9 (0.5-1.4)
**CSW**					
No	1.0	NS	1.0	NT	NS
Yes, in <6 months	**0.8 (0.7-0.9)**		**0.8 (0.6-0.9)**		
Unknown	**0.4 (0.3-0.6)**		**0.3 (0.1-0.5)**		
**Client of CSW**					
No	1.0	1.0	1.0	NS	NS
Yes, in <6 months	**0.6 (0.5-0.7)**	**0.7 (0.5-0.8)**	**0.6 (0.5-0.8)**		
Unknown	1.4 (1.0-2.1)	**0.7 (0.5-0.9)**	1.7 (0.9-3.2)		
**No of partners in < 6 months**					
0-1	1.0	1.0	1.0	1.0	1.0
2-10	**1.5 (1.4-1.6)**	**1.5 (1.3-1.6)**	**1.8 (1.6-2.0)**	0.9 (0.7-1.0)	**1.3 (1.1-1.5)**
>10	**1.9 (1.8-2.1)**	**1.7 (1.6-1.9)**	**2.4 (2.1-2.7)**	0.9 (0.7-1.1)	**1.7 (1.4-2.1)**
Unknown	**1.6 (1.5-1.7)**	1.5 (1.4-1.7)	**1.9 (1.7-2.2)**	**0.7 (0.6-0.9)**	**1.3 (1.1-1.6)**
**Notified**					
No	1.0	1.0	1.0	1.0	1.0
Yes	**2.2 (2.1-2.3)**	**1.9 (1.8-2.1)**	**2.3 (2.1-2.4)**	**1.9 (1.6-2.1)**	**2.2 (1.9-2.4)**
Unknown	**1.1 (1.0-1.2)**	1.0 (1.0-1.1)	**1.3 (1.1-1.4)**	**1.2 (1.0-1.4)**	0.8 (0.7-1.0)

NS: Not significant. NT: Not tested in multivariate model (p >0.20 in univariate model).

In bold: significant (p < 0.01); due to rounding into 1 decimal 1.0 is not always significant.

## Discussion

During 2006–2012, the number of STI consultations by MSM in Dutch STI clinics increased steadily in both age groups, whereby highest positivity rates and specific risks were observed for younger MSM. Of all MSM clinic visitors aged 15–24 years, 19% tested positive for at least one STI, and this positivity rate increased significantly since 2007, driven by higher gonorrhoea positivity rates. Among MSM 25 years and older, overall positivity rate was 19%, but decreased since 2006 and remained stable since 2009. Positivity rates for syphilis decreased in both young and older MSM, and in older MSM HIV positivity rate also decreased significantly. Specific ethnic groups were at higher risk for testing positive for any STI, as were those reporting exclusively having sex with men, those with a history of STI, those reporting having multiple sex partners and those notified for an STI.

Among MSM younger than 25 years, Surinamese and Latin American backgrounds were stronger associated with testing positive for an STI, as well as the number of sexual partners and being notified for an STI, compared to older MSM. Young MSM involved in CSW also tested more often positive than those not involved in CSW, but no such effect was observed among older MSM. Having a previous HIV test was protective among older MSM, but not among younger MSM.

The increasing and high STI positivity rates in young MSM are cause for great concern. This is mainly driven by persistently high positivity rates for chlamydia and an increasing positivity rate for gonorrhoea. The latter is especially worrisome with regard to the increasing problem of antimicrobial resistance with the risk of gonorrhoea becoming untreatable [13].

Special attention needs to be paid towards counselling of sexually risk taking in young MSM, since adolescents and young adults are exploring their sexuality and disclosure regarding sexual identity and sexual behaviour may be limited [14]. Young MSM use the internet and social media among others to search for sex partners and for information regarding safe sex and STI [15]. This provides us with the opportunity of developing new web-based and multi-media interventions for prevention in order to reach young MSM. The effective use of such new media in establishing new prevention activities, including e-health interventions or placing banners and STI information on websites or other online media which are frequently used by MSM needs to be explored and the effectiveness of such interventions should be assessed.

Current data show that non-Dutch MSM in both age groups are also at high-risk testing positive for an STI. A recent Dutch survey pointed out that young migrant MSM are hardly reached by current prevention and intervention programmes [15]. Current HIV and STI prevention programs targeted at migrant MSM should be strengthened to increase their awareness of and knowledge on STI, in particular around HIV, and reduce stigma around homosexuality. In addition, ‘mainstream’ interventions focused at reaching MSM should be culturally sensitive to attract a broader audience.

The current study shows that within the population of young MSM, those involved in commercial sex were at significantly higher risk for testing positive for an STI than those not involved. Although the group of MSM visiting an STI clinic who are involved in CSW is only a small group, targeted health education to encourage safer sex work is needed to improve both the health of the sex workers and their clients. This is important since sex workers have many (unsafe) sexual contacts and therefore STIs can be transmitted more quickly. Little is known about the determinants of (un)safe sex of MSM involved in commercial sex and research on this would be necessary in order to develop effective interventions targeted at this specific group.

The loss of fear regarding HIV transmission due to better treatment options for HIV infection by combination antiretroviral therapy (cART) may play a role in the explanation for the persistent high rates of STI in especially young MSM and the outcome that no protective effect of previous HIV testing was found in young MSM [16-18]. The so-called ‘post-aids generation’ did not experience the dying, multiple losses and fear for infection during the first decennia of the HIV epidemic. Stolte et al. [18] report an increase in MSM attending STI clinics and a rise in infection rates coinciding with the introduction of cART. Data from an online survey among Dutch MSM showed a significant increase in unprotected anal intercourse with a casual partner from 33% in 2009 to 36% in 2011 [15], indicating high-risk behaviour. Since 2011, condom use with the last sex partner (casual or steady) is registered for all STI clinic attendees. In both age groups, 61% of MSM reported not having used a condom during their last sexual contact with a casual partner (data not shown).

Our study included only data from STI clinics and extrapolation to the general population and MSM community should be done with caution, since the STI clinics are facilities set up for high risk groups, in addition to regular primary health care. Most people in the Netherlands consult their general practitioner (GP) for STI testing [19,20]. However, two recent Dutch studies comparing data from GPs with STI clinics, showed that STI clinic visitors are more often from high-risk groups, like MSM [20,21]. Trienekens et al. showed that only 9% of consultations in GPs concerned MSM, compared to more than one third in STI clinics [21]. Also, results from a recent survey (2011) in the Netherlands showed that of the MSM who were tested for HIV in the past six months, 60% was tested at an STI clinic, compared to 28% at their GP [15]. These results suggest that MSM who want to get tested perceived a low threshold to access STI clinics. Another limitation of current study is that no information is available about the unique number of MSM attending the STI clinics, but only about the number of consultations in MSM. This might influence the different outcomes.

## Conclusion

In conclusion, MSM visiting STI clinics remain a high-risk group for STI infections and transmission, but are not a homogenous risk group. While in MSM aged older than 25 years, STI positivity is decreasing, a significant increase in positivity rate was found in young MSM. Special sub-groups, like young migrant MSM and young MSM sex workers, do also need to be targeted specifically to prevent further transmission of STI.

### Key messages

● MSM visiting STI clinics remain a high-risk group

● An increase in STI positivity rate was found in young MSM

● Counselling and reaching specific MSM sub-groups at highest and increasing risk remains very important

## Competing interests

The authors declare that they have no competing interests.

## Authors' contributions

The study was designed by FK, BB, EV, WZ and MS. BB and MS supervised data analysis. FKB analysed the data and wrote the first draft of the article. All authors read and approved the final manuscript.

## Authors’ information

Dutch STI clinics: A van Daal (East), AP van Leeuwen (North-Holland Flevoland), F. de Groot (North), CJPA Hoebe (Limburg), K Hulshof (Utrecht), A van Camerijk (South-Holland North), JCAM van de Sande (Zeeland-Brabant), V Wieërs (South-Holland South).

## Supplementary Material

Additional file 1: Table S1 Results for the univariate regression analyses for any STI and in consultations in young MSM (<25 years) and older MSM (≥25 years) visiting STI clinics, the Netherlands, 2006–2012. Table S1 is presenting the univariate odds ratios (ORs) for the associoation between variables included in the study and the outcome of having any STI. Results are presented separately for young MSM (<25 years) and older MSM (≥25 years) who visited an STI clinic in the Netherlands between 2006 and 2012. Click here for file
