# Supplementary material for: Increasing sexually transmitted infection rates in young men having sex with men in the Netherlands, 2006–2012
Source: Emerg Themes Epidemiol. 2014 Aug 28;11:12. doi: 10.1186/1742-7622-11-12 (PMC4147385; doi:10.1186/1742-7622-11-12)
Supplement: Additional file 1: Table S1 — Results for the univariate regression analyses for any STI and in consultations in young MSM (<25 years) and older MSM (≥25 years) visiting STI clinics, the Netherlands, 2006–2012. Table S1 is presenting the univariate odds ratios (ORs) for the associoation between variables included in the study and the outcome of having any STI. Results are presented separately for young MSM (<25 years) and older MSM (≥25 years) who visited an STI clinic in the Netherlands between 2006 and 2012. [file 1742-7622-11-12-S1.docx]

*Table S1. Results for the univariate regression analyses for any* *STI and in consultations in young MSM (<25 years) and MSM aged25 years or older visiting STI clinics, the Netherlands, 2006-2012*

|  | **Any STI in young MSM (<25 years)**  Univariate OR (95% CI) | **Any STI in young MSM (≥25 years)**  Univariate OR (95% CI) |
| --- | --- | --- |
|  |  |  |
| **Year of consult** | **1.04 (1.0-1.1)** | **0.96 (0.9-1.0)** |
| **Age, years** |  |  |
| 15-19 | 1.0 |  |
| 20-24 | **1.2 (1.1-1.3)** |  |
| 25-34 |  | 1.0 |
| 35-44 |  | **0.9 (0.9-1.0)** |
| 45-54 |  | **0.8 (0.8-0.9)** |
| ≥55 |  | **0.6 (0.6-0.7)** |
| **Sexual behaviour** | |  |
| Sex with both men and women | 1.0 | 1.0 |
| Sex with men only | **1.2 (1.1-1.3)** | **1.6 (1.5-1.7)** |
| **Ethnicity** |  |  |
| Dutch | 1.0 | 1.0 |
| Surinam/ Antilles | **1.9 (1.7-2.3)** | **1.5 (1.4-1.7)** |
| Turkey/Morocco | 1.2 (0.9-1.5) | **1.3 (1.1-1.4)** |
| Eastern Europe | **1.9 (1.5-2.3)** | **1.4 (1.2-1.5)** |
| SS Africa | **1.5 (1.0-2.1)** | **1.6 (1.3-1.9)** |
| Latin America | **2.1 (1.7-2.6)** | **1.3 (1.2-1.5)** |
| Asia | 1.1 (0.9-1.4) | **1.4 (1.3-1.5)** |
| Other | **1.3 (1.1-1.5)** | **1.1 (1.0-1.2)** |
| **Previous HIV test** | |  |
| No | 1.0 | 1.0 |
| Yes, negative | **1.3 (1.2-1.4)** | **0.9 (0.9-1.0)** |
| Unknown | 0.8 (0.6-1.1) | **0.8 (0.7-0.9)** |
| **Previous STI** |  |  |
| No | 1.0 | 1.0 |
| Yes | **1.9 (1.7-2.1)** | **1.7 (1.6-1.8)** |
| Unknown | 0.9 (0.8-1.0) | **1.2 (1.1-1.3)** |
| **CSW** |  |  |
| No | 1.0 | 1.0 |
| Yes, in <6 months | **1.9 (1.6-2.3)** | 0.9 (0.8-1.1) |
| Unknown | **0.4 (0.2-0.7)** | **0.5 (0.4-0.7)** |
| **Client of CSW** |  |  |
| No | 1.0 | 1.0 |
| Yes, in <6 months | 1.0 (0.7-1.5) | **0.5 (0.4-0.6)** |
| Unknown | 0.4 (0.2-0.7) | **0.6 (0.5-0.8)** |
| **No of partners in < 6 months** | |  |
| 0-1 | 1.0 | 1.0 |
| 2-10 | **1.5 (1.3-1.7)** | **1.4 (1.3-1.5)** |
| >10 | **2.9 (2.4-3.4)** | **1.8 (1.7-1.9)** |
| Unknown | **1.4 (1.2-1.6)** | **1.6 (1.5-1.7)** |
| **Notified** |  |  |
| No | 1.0 | 1.0 |
| Yes | **2.9 (2.6-3.2)** | **2.2 (2.1-2.3)** |
| Unknown | 1.0 (0.9-1.1) | **1.3 (1.3-1.4)** |

NS: Not significant. In bold: significant (p<0.01); due to rounding into 1 decimal 1.0 is not always significant
